# Dynamic m^6^A Modification Landscape During the Egg Laying Process of Chickens

**DOI:** 10.3390/ijms26041677

**Published:** 2025-02-16

**Authors:** Yushi Zhang, Yida Chen, Haigang Ji, Yufang Niu, Liyang He, Wentao Wang, Tong Yu, Ruili Han, Yadong Tian, Xiaojun Liu, Xiangtao Kang, Hanfang Cai, Zhuanjian Li

**Affiliations:** 1 College of Animal Science and Technology, Henan Agricultural University, Zhengzhou 450046, China; zys040828@163.com (Y.Z.); 13253799792@163.com (Y.C.); xingkongjhg@163.com (H.J.); 13462940651@163.com (Y.N.); 18238497653@139.com (L.H.); m15538222009@163.com (W.W.); yu1015tong@163.com (T.Y.); rlhan@126.com (R.H.); ydtian111@163.com (Y.T.); xjliu2008@hotmail.com (X.L.); xtkang2001@263.net (X.K.); 2 Key Laboratory of Livestock and Poultry Resources (Poultry) Evaluation and Utilization, Ministry of Agriculture and Rural Affairs, Henan Agricultural University, Zhengzhou 450046, China; 3 International Joint Research Laboratory for Poultry Breeding of Henan, Henan Agricultural University, Zhengzhou 450046, China

**Keywords:** m^6^A methylation, methylated RNA immunoprecipitation sequencing, follicle, egg production, Gushi chicken, ovary

## Abstract

RNA N6-methyladenosine (m^6^A) is one of the most common and widespread reversible epigenetic modifications of mRNAs, and m^6^A has been shown to play a positive role in regulating follicular development. However, the role of RNA m^6^A methylation in chicken ovaries and egg production has not been fully studied. In this study, we comprehensively analyzed the m^6^A transcriptome profiles of high- and low-yield Gushi chickens at 43 weeks of age (43 w). We found that m^6^A modification differed between the two groups. The m^6^A peak was positively correlated with the gene expression level, indicating that m^6^A may play an important role in regulating chicken egg production. In total, 9008 and 15,415 m^6^A peaks were separately identified in the two groups, including 2241 differential m^6^A peaks. In addition, seven candidate genes related to egg laying that were significantly enriched in the KEGG pathway related to ovary development and egg laying were identified. In summary, we constructed the first m^6^A modification map of ovarian tissue of Gushi chickens, and the differences in egg laying in 43 w Gushi chickens may originate from the effect of RNA methylation on the expression of egg-related genes. These findings provide new insights into the regulatory mechanisms of m^6^A methylation during egg production in Gushi chickens.

## 1. Introduction

With the development of animal husbandry and improvements in living standards, the demand for meat, eggs, and dairy products has increased. Owing to their excellent protein content and low prices, poultry eggs have become indispensable sources of protein in people’s lives. The Gushi chicken is an indigenous Chinese dual-purpose chicken used for both eggs and meat and has the characteristics of producing more eggs, having a large egg volume, having a thick eggshell, and being resistant to storage and transportation. However, as a local chicken breed, Gushi chickens have a relatively low egg production rate. Therefore, improving the egg production performance of Gushi chickens has become an important goal.

The ovary is the main reproductive organ of female animals and plays an important role in follicular development and hormone secretion. Previous studies have shown that the egg production performance of laying chickens is closely related to ovary and follicular development and that the development status of the follicles has a direct effect on the egg numbers of chickens [1]. Successive follicle selection is important for egg production and reproductive performance in chickens [2]. In addition, some epigenetic studies have shown that RNA N6-methyladenosine (m^6^A) methylation and DNA methylation regulate follicular development in chickens [3,4].

m^6^A is one of the most abundant and highly conserved forms of posttranscriptional modification in eukaryotes and is widely found in mRNAs, tRNAs, rRNAs, miRNAs, and lncRNAs [5]. m^6^A reportedly plays an active role in posttranscriptional regulation, including the regulation of mRNA stability, splicing, translation, and gene expression [6]. The m^6^A modification process is dynamically reversible and relies on the regulation of multiple proteins, including m^6^A methyltransferases (writers), demethylation enzymes (erasers), and methylation recognition proteins (readers) [7]. In addition, m^6^A is involved in various physiological processes in mammals. In the female reproductive system, m^6^A modifications regulate multiple stages of oogenesis and pathogenesis in a variety of female reproductive diseases [8]. In avian animals, such as chickens, m^6^A modification has a significant effect on follicle selection [4]. m^6^A has been identified as a key factor in the sexual maturation process of male yak testes [9] and the follicular development process of female yaks [10]. In addition, m^6^A modification plays a regulatory role in the development of pig testes [11]. Notably, numerous m^6^A-related proteins participate in the development of mouse oocytes and early embryos by regulating the conversion of material mRNAs [12]. The m^6^A methyltransferase METTL3 is involved in mammalian spermatogenesis and plays key roles in the m^6^A content of sperm RNA [13].

Previous studies on m^6^A have focused mostly on humans, plants, and mammals, with few reports on m^6^A in laying chickens. The mechanism of action of m^6^A remains poorly understood. With the development of high-throughput sequencing technology and the deepening of epigenetics research, researchers have constructed various animal and plant maps using methylated RNA immunoprecipitation sequencing (MeRIP-seq) technology. Therefore, to explore the dynamic changes exhibited by m^6^A modifications during chicken egg production and their effects on ovary development, we constructed an m^6^A transcriptome profile of the ovaries of 43 w high- and low-yield Gushi chickens. This study provides a theoretical reference for revealing the regulatory mechanism of m^6^A modification in the egg production process of Gushi chickens, which may be highly important for improving egg production performance.

## 2. Results

### 2.1. Egg Production, Ovarian Weight, and Colorimetric Results

**Figure 1:**
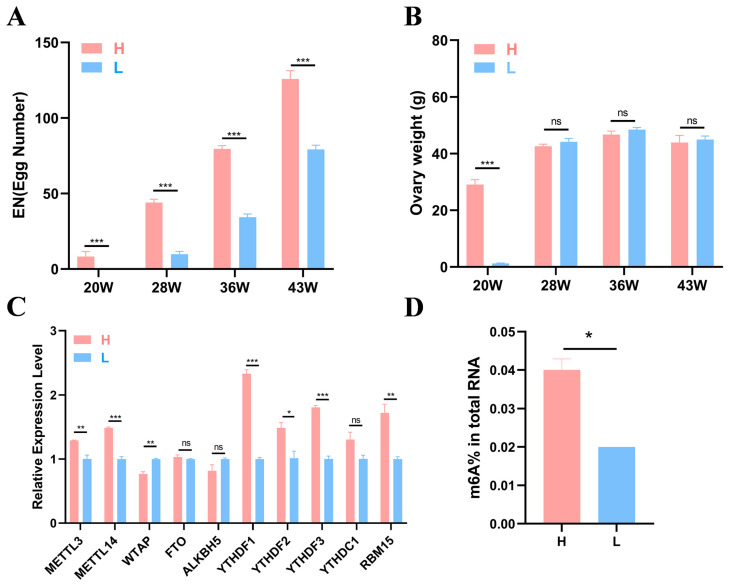
Production data and experimental results. (**A**,**B**) Statistical results of egg number and ovary weight of high- and low-yield Gushi chickens at 20, 28, 36, and 43 weeks of age (*n* = 6). (**C**) Gene expression levels of 10 common m^6^A methylases in the H and L groups of 43-week-old Gushi hens. (**D**) The significance of the differences between the egg numbers and ovary weights of the H group and L group was determined using Student’s *t* test (* *p* < 0.05; ** *p* < 0.01; *** *p* < 0.001, ns *p* > 0.05).

Six healthy individuals from the high-yield (H) and the low-yield (L) groups were randomly selected at each time point (20, 28, 36, and 43 weeks) for ovarian weight measurement and sample collection. Details for the egg production and ovarian weight are shown in Figure 1A,B. The results revealed that at 20, 28, 36, and 43 weeks of age, the egg number (EN) of the H group was significantly greater than that of the L group at each stage (*p* < 0.001), and the EN of both groups tended to increase (Figure 1A). At 20 weeks, a significant difference in ovary weight was noted between the two groups (*p* < 0.001), whereas no differences were observed at other stages (Figure 1B).

To further investigate whether m^6^A modification can affect egg production in Gushi chickens, we detected the expression levels of 10 m^6^A enzymes corresponding genes (*METTL3*, *METTL14*, *WTAP*, *FTO*, *ALKBH5*, *YTHDF1*-*3*, *YTHDC1*, and *RBM15*) in the ovarian tissues of two groups of Gushi chickens at 43 w using quantitative real-time PCR (qRT–PCR). The results revealed that the *YTHDF2* expression level in group H was significantly greater than that in group L (*p* < 0.05). *WTAP* expression levels in group L were significantly greater than those in group H (*p* < 0.01, Figure 1C), and *METTL3* and *RBM15* expression levels in group H were also significantly greater than those in group L (*p* < 0.01). Differences in *METTL14*, *YTHDF1*, and *YTHDF3* expression levels were noted between the H and L groups (*p* < 0.001). The test results revealed significant differences in the expression of these methylases between the different groups. To verify whether there was a m^6^A modification difference between the two groups, we conducted colorimetric experiments. The results revealed a significant difference between groups L and H, with group H having a greater degree of methylation compared with group L (*p* < 0.05, Figure 1D).

### 2.2. Sequencing Quality Control and Reference Genome Alignment

**Figure 2:**
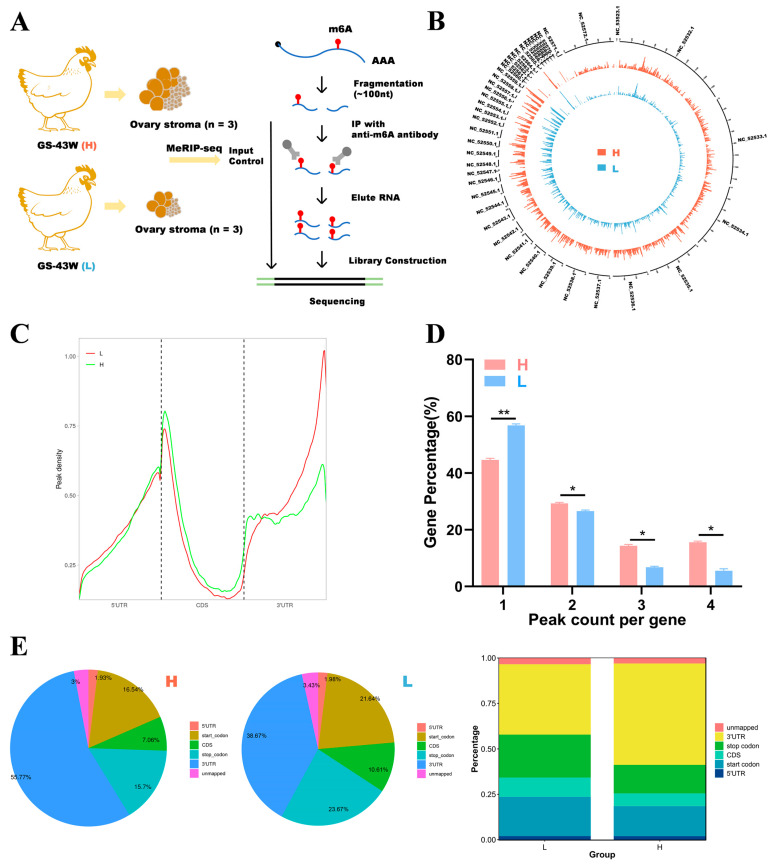
Research on m^6^A modification profiles in the ovaries of Gushi chickens. (**A**) Ovary tissue was collected from 43-week-old Gushi chickens in group H (*n* = 3) and group L (*n* = 3) for MeRIP-seq. (**B**) The distribution of m^6^A peak sites in the *Gallus gallus* (chicken) genome was obtained from two different samples. The outermost circles represent the distribution of chromosomes in the genome, with red circles representing group H and blue circles representing group L. (**C**) Density distribution of the m^6^A peaks in H and L transcripts in the different gene structures. (**D**) Statistical graph of the peak numbers of genes in the L and H groups. The difference between the peak numbers of genes in the L and H groups was detected using Student’s *t* test (* *p* < 0.05; ** *p* < 0.01;). (**E**) Distribution of m^6^A peaks in the L and H groups.

We selected three chickens from each of the 43-week-old Gushi chickens in the H and L groups and performed MeRIP sequencing on the ovarian tissues. The unqualified sequences in the raw data were screened using Fastp to obtain clean data. Clean data were analyzed using MeRIP-seq and RNA sequencing (RNA-seq) to obtain the m^6^A transcriptome profile of the ovary tissue of the 43-week-old Gushi chickens. The MeRIP-seq process is shown in Figure 2A. The percentages of valid data in each group of samples based on mass ≥ 20 (sequencing error rate less than 0.01), mass ≥ 30 (sequencing error rate less than 0.001), and GC content are shown in Supplementary Table S1. The immunoprecipitation (IP) samples from the ovaries of the chickens in the L and H groups were recorded as L_IP and H_IP, respectively. In the RNA-seq library, these samples are recorded as L_input and H_input. Supplementary Table S2 shows the unique mapped reads. MeRIP-seq of the L and H groups generated averages of 57,339,748 and 60,948,454 raw reads and 56,399,912 and 59,904,270 pure reads, respectively. The percentage of effective reads exceeded 98%. We calculated the distribution of reads in the reference genome and categorized the reads into exon, intron, and intergenic regions, as shown in Supplementary Figure S1. Most of the reads were mapped to exon regions.

### 2.3. Analysis of m^6^A Modifications in the Transcriptome

As shown in Figure 2B, the distributions of m^6^A modification sites in the chicken genome were generally consistent between the two groups. As shown in Figure 2C, the density distributions of the m^6^A peaks were similar across all the groups, but the specific distributions were not the same. To understand the extent of m^6^A modification in differentially methylated genes (DMGs), we analyzed the distribution of m^6^A peaks in each gene and found that almost half of the genes in the two groups presented only one m^6^A modification peak and that most genes presented 1–3 m^6^A peaks (Figure 2D). To investigate the importance of m^6^A in transcripts, the distribution of m^6^A peaks in mRNAs was explored in conjunction with the chicken reference genome. The transcripts were categorized into 5′ untranslated regions (5′ UTRs) near the start codon, 3′ untranslated regions (3′ UTRs) near the stop codon, and coding DNA sequence (CDS) regions. As shown in Figure 2E, the m^6^A peaks in the H group were enriched mainly in 3′ UTRs, followed by start codons, stop codons, CDS regions and 5′ UTRs. In the L group, the m^6^A peaks were enriched in 3′ UTRs, followed by stop codons, start codons, CDS regions and 5′ UTRs. In addition, more m^6^A peaks were found in 3′ UTRs in the H group compared with the L group. The L group presented more m^6^A peaks enriched at start and stop codons compared with the H group.

### 2.4. Analysis of Differentially Methylated Genes

**Figure 3:**
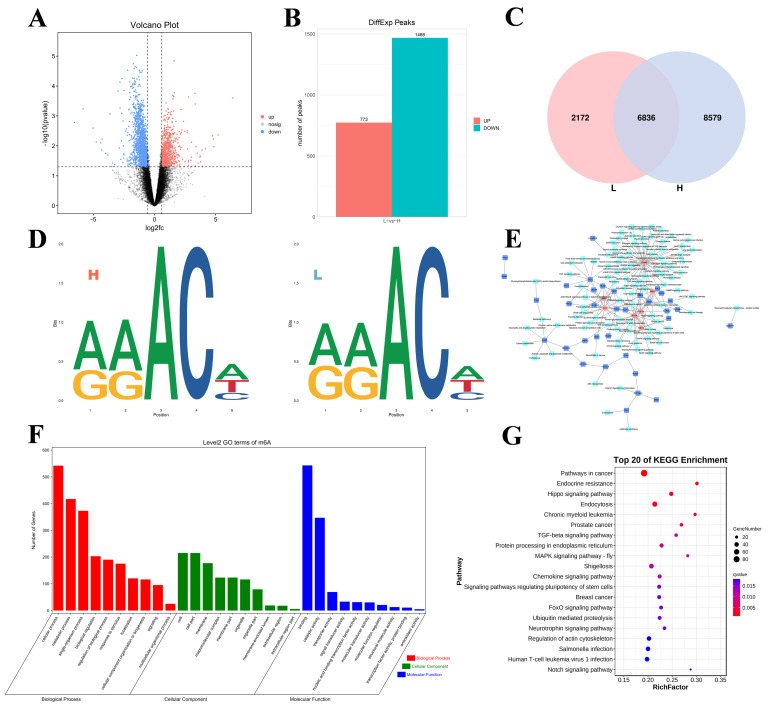
Overview of the differential methylation peaks in the L vs. H comparison groups. (**A**) Volcano plot of the DMG expression results (the dotted line signifies the threshold for statistical significance). (**B**) Number of up- and downregulated DMPs. (**C**) The number of common and specific m^6^A peaks in the L and H groups. (**D**) The motif sequence “RRACH” was identified in the L and H. (**E**) Pathways and co-differential genes network diagram. (**F**,**G**) GO enrichment terms and KEGG analysis of DMGs.

The volcano diagram shows the overall distribution of differentially methylated peaks (DMPs; Figure 3A). Specifically, 2241 DMPs in 2068 DMGs were identified in the H group relative to the L group. A total of 773 peaks presented increased expression levels (corresponding to 786 genes with upregulated m^6^A levels), and 1468 peaks presented decreased expression levels (corresponding to 1372 genes with downregulated m^6^A levels), as shown in Figure 3B. In addition, we detected 8579 specific m^6^A peaks in the H group and 2172 specific peaks in the L group (Figure 3C), reflecting the significant differences in m^6^A modifications between the groups. We performed motif prediction using samples from both groups, as shown in Figure 3D. Seven genes were screened using gene network mapping. These genes included *WNT4*, *FN1*, *FGF16*, *CRISPLD2*, *AMH*, *HEY1*, and *GNAQ* (Figure 3E).

In addition, GO and KEGG enrichment analyses of all the DMGs were performed to explore the important roles of m6A modifications in egg production. The GO analysis revealed that all the DMGs were involved in cellular processes, metabolic processes, and single organism processes (ontology: biological processes); cell, cell part, and cell mem-brane processes (ontology: cellular components); and binding, catalytic activity, and transporter activity (ontology: molecular function) (Figure 3F). KEGG enrichment analysis revealed that the DMGs were associated with pathways associated with cancer, the MAPK signaling pathway-fly, the FoxO signaling pathway, and the Notch signaling pathway (Figure 3G).

### 2.5. RNA-Seq Identification of Differentially Expressed Genes

**Figure 4:**
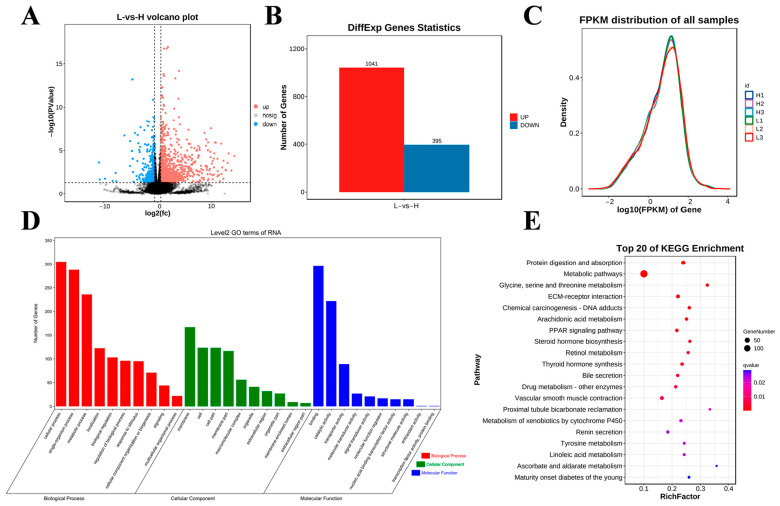
Differential expression analysis of RNA-seq data among the studied groups. (**A**) Volcano diagram of DEG expression data (the dotted line signifies the threshold for statistical significance). (**B**) Number of up- and downregulated DEGs. (**C**) Gene expression abundance distribution map. (**D**,**E**) GO enrichment terms and KEGG analysis of DEGs.

These samples were also analyzed using RNA-seq, and the generated volcano dia-gram shows the general distribution of the differentially expressed genes (DEGs; Figure 4A). In this study, a total of 19,902 genes were identified in the two groups, and 1436 genes were differentially expressed in the H group compared with the L group (1041 upregulated genes and 395 downregulated genes; *p* < 0.05 and |fold change (FC)| > 1.5; Figure 4B). The gene expression abundance and density data are shown in Figure 4C. To further reveal the functions of the DEGs, we conducted GO and KEGG enrichment analyses. The results from the GO enrichment analysis of the DEGs are shown in Figure 4D. Among the biological processes, the DEGs were enriched mainly in cellular processes, single organism processes, metabolic processes, biological regulation, and regulation of biological processes. In the cellular component category, the DEGs were significantly correlated mainly with membrane, cell, cell part, and membrane part. In the molecular function category, the DEGs were involved mainly in binding activity, catalytic activity, and transporter activity. The KEGG enrichment analysis revealed that the DEGs were significantly enriched in protein digestion and absorption, the PPAR signaling pathway, thyroid hormone synthesis, and steroid hormone biosynthesis (Figure 4E).

### 2.6. Joint Analysis of MeRIP-Seq and RNA-Seq Data

**Figure 5:**
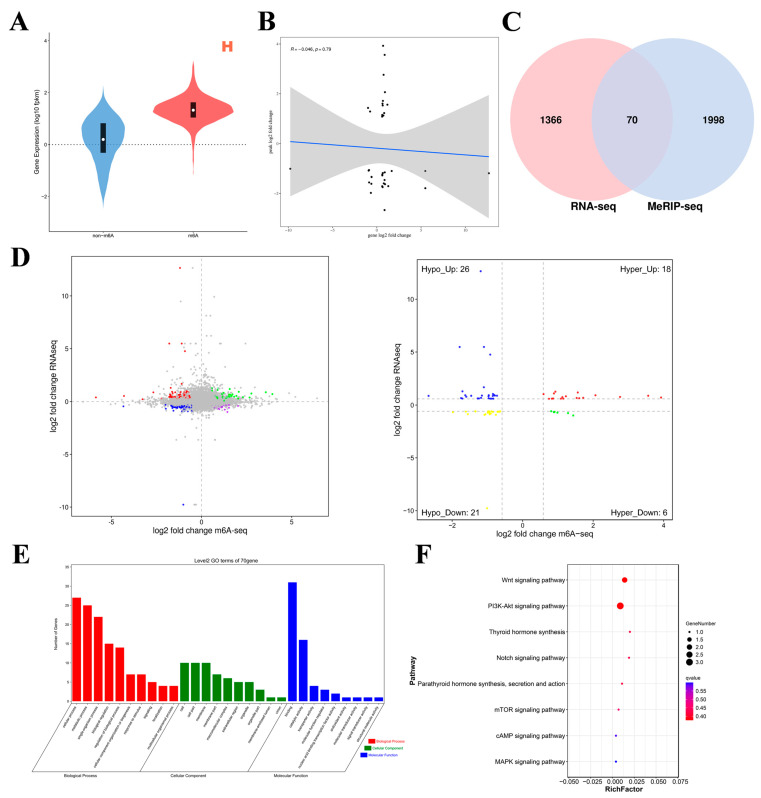
Joint analyses of m^6^A-seq and RNA-seq data. (**A**) Violin plot of expression with/without m^6^A-modified genes. (**B**) Scatter plot of gene expression levels and peak enrichment multiples (the gray area represents the confidence interval. Specifically, it represents the uncertainty range of regression line estimation). (**C**) Wayne map of shared differentially expressed genes. (**D**) L vs. H quadrant diagram (the dotted line signifies the threshold for statistical significance; the four colors signify four distinct scenarios: mRNA exhibiting concurrent upregulation or downregulation with the m^6^A group, or alternatively, one group demonstrating upregulation while the other shows downregulation). (**E**,**F**) GO enrichment terms and KEGG analysis of co-differentially expressed genes (intersecting genes of DMGs and DEGs).

To investigate the relationship between m^6^A modification and gene expression, we conducted MeRIP-seq and RNA-seq association analyses. For each group of samples, genes were categorized according to the presence or absence of m^6^A modifications, and the corresponding gene expression trend within the group was observed to analyze the effect of m^6^A modifications on gene expression. Figure 5A shows that the overall expression level of mRNAs with m^6^A modifications in Group H was greater than that of mRNAs without m^6^A modifications, and the same finding was true for Group L (Supplementary Figure S2). Therefore, m^6^A potentially positively regulates mRNA expression. To analyze the relationship between expression levels and peak enrichment, we generated a scatter plot of gene expression levels and peak enrichment. As shown in Figure 5B, genes with relatively high expression levels presented relatively low peak enrichment. Therefore, we preliminarily concluded that a negative correlation exists between m^6^A methylation levels and gene expression levels. The generated four-quadrant diagram and Venn diagram (Figure 5C,D) revealed a total of 70 DMGs in the L group relative to the H group, including 24 hypermethylated DMGs (18 upregulated mRNAs and 6 downregulated mRNAs) and 47 hypomethylated DMGs (26 upregulated mRNAs and 21 downregulated mRNAs). In the left panel of Figure 5D, each point represents a gene. Green points indicate genes where both m^6^A peak and mRNA expression are consistently upregulated. Red points represent genes where mRNA expression is upregulated while the m^6^A peak expression is downregulated. Blue points indicate genes where both m^6^A peak and mRNA expression are consistently downregulated. Purple points represent genes where mRNA expression is downregulated while the m^6^A peak expression is upregulated. Gray points indicate genes with no significant difference between groups (transcriptome FDR > 0.05 and m^6^A *p*-value > 0.05). The right panel of Figure 5D is similar to the left panel but with different coloring, as explained for the left panel.

GO analysis of the co-differentially expressed genes was performed (Figure 5E). In terms of molecular function, these genes were significantly correlated with binding activity, catalytic activity, and transporter activity. In terms of biological processes, these genes significantly participated in cellular processes, metabolic processes, single organism processes, biological regulation, and regulation of biological processes. In the cell component category, these genes were related mainly to cell parts, modules, and cells. KEGG enrichment analysis results (Figure 5F) revealed that these genes were significantly enriched in the PI3K–Akt signaling pathway; thyroid hormone synthesis; the Notch signaling pathway; parathyroid hormone synthesis, secretion, and action; the mTOR signaling pathway; the cAMP signaling pathway; and the MAPK signaling pathway. These significantly enriched pathways are related to ovary hormone secretion and follicular development. These results indicate that m^6^A modification is involved in various physiological activities of the ovaries during egg production in Gushi chickens.

**Figure 6:**
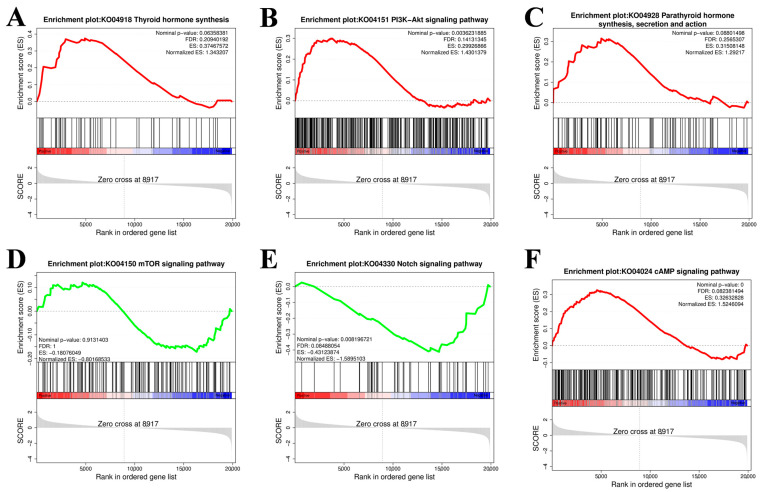
Gene set enrichment analysis (GSEA). (**A**) Thyroid hormone synthesis. (**B**) PI3K-Akt signaling pathway. (**C**) Parathyroid hormone synthesis. (**D**) mTOR signaling pathway. (**E**) Notch signaling pathway. (**F**) cAMP signaling pathway. The gray area in the figure represents a point in the gene list where the Enrichment Score (ES) crosses a zero value. The dashed line usually represents the Zero cross point of the enrichment score.

The gene set enrichment analysis (GSEA) results were consistent with the abovementioned KEGG enrichment results for the PI3K–Akt signaling pathway, thyroid hormone synthesis pathway, Notch signaling pathway, parathyroid hormone synthesis, secretion and action, mTOR signaling pathway, cAMP signaling pathway, and MAPK signaling pathway (Figure 6).

### 2.7. Results of MeRIP-qPCR and qRT–PCR Experiments

**Figure 7:**
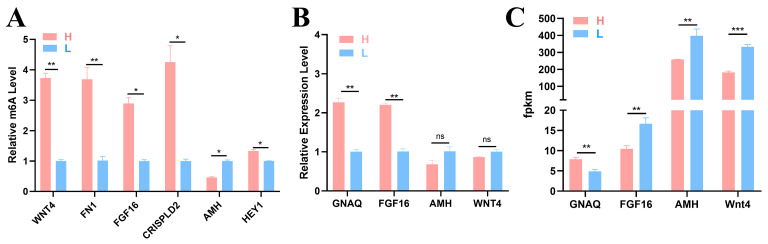
Experimental results. (**A**) Methylase gene levels were detected using MeRIP-qPCR. (**B**) The relative mRNA expression level was detected using qRT–PCR. (**C**) Gene change levels based on RNA-Seq data. The significance of the difference between the gene expression levels of the H group and L group was determined by Student’s *t* test (* *p* < 0.05; ** *p* < 0.01; *** *p* < 0.001, ns *p* > 0.05).

The following seven key node genes were identified by conducting a network diagram analysis of the candidate genes: *WNT4*, *FN1*, *FGF16*, *CRISPLD2*, *AMH*, *HEY1*, and *GNAQ* (Figure 3E). To verify the accuracy of the sequencing results, six DMGs (*WNT4*, *FN1*, *FGF16*, *CRISPLD2*, *AMH*, and *HEY1*) and four DEGs (*GNAQ*, *FGF16*, *AMH*, and *WNT4*) were selected for detection via MeRIP–qPCR and qRT–PCR. The methylation levels of the six genes shown in Figure 7A differed between the two groups, indicating the presence of m^6^A methylation in the ovary tissues of the two groups of 43 w Gushi chickens. The gene methylation level results were consistent with the sequencing results, confirming the authenticity and reliability of the sequencing results (Figure 7B,C).

## 3. Discussion

In the modern poultry industry, egg production is essential and has a certain economic and reproductive value, and egg production performance is also an important factor affecting the poultry industry. The ovary is the main reproductive organ of female animals and plays an important role in ovum laying and hormone secretion. Ovary development in chickens is a dynamic process regulated by multiple factors, and the laying performance of chickens strongly depends on ovary function. The development of ovary follicles is a key factor affecting the egg production traits of poultry. Follicles are the basic functional unit of ovaries. In female poultry, primordial follicles gradually form after birth, and the number of primordial follicles increases continuously with ovarian development. However, a large part of this regulatory process has not been fully elucidated. The molecular mechanisms of chicken ovary development or follicle selection are relatively complex, and m^6^A modification plays an important role in follicle selection [4].

With the continuous development of science and technology, RNA methylation has become a hot topic in the study of epigenetics. m^6^A regulates RNA stability, variable splicing, out-of-nucleus translocation, degradation, selective translation, and translation rates, and is also related to RNA stability [14]. It affects RNA stability, which subsequently determines whether RNA is degraded [15]. Currently, m^6^A-related research has involved multiple animals. In 2019, [4] constructed a transcriptome-wide m^6^A methylation profile of chicken ovaries during follicle selection, and the results revealed that pre-hierarchical and hierarchical follicles presented greater m^6^A enrichment near the start and stop codon regions. Most of the methylation peaks in yaks are concentrated in the stop codon as well as the 3′ UTR and 5′ UTR regions [9]. The results of the present study are consistent with the findings of the abovementioned studies, demonstrating that m^6^A methylation is highly enriched near the 3′ UTR, start codon, and stop codon regions. Thus, these results suggest that the overall distributions of m^6^A methylation peaks are highly similar among species. In addition, our data revealed that the high-yield group presented high levels of m^6^A methylation and that the m^6^A levels of the mRNAs in the two groups significantly differed, suggesting that m^6^A may affect egg production ability. For example, [10] reported that m^6^A-modified genes play key regulatory roles in female yaks’ follicular growth and development. According to MeRIP sequencing results from oocytes, m^6^A modifications play a coordinating role in RNA stabilization during mouse follicular development and oocyte growth [12].

In this study, we investigated the dynamic changes in m^6^A methylation in the ovary transcriptomes of high- and low-yield Gushi chickens during sexual maturity. Many methylation peaks were detected in the transcriptome of Gushi chicken ovary tissue, and 2241 m^6^A peaks, and 2068 DMGs were identified. GO and KEGG enrichment analyses and GSEA of 70 DMGs identified using both omics methods revealed that the DMGs have potential regulatory functions in ovary development. These genes are involved in various important pathways, such as the PI3K–Akt signaling pathway, the Wnt signaling pathway, the mTOR signaling pathway, the cAMP signaling pathway, the insulin secretion pathway, the MAPK signaling pathway, and the Notch signaling pathway. Among them, the pathway with the most enriched genes was the PI3K–Akt signaling pathway.

Phosphatidylinositol 3-kinase (PI3K) signaling is a fundamental pathway for the regulation of cell proliferation, survival, migration, and metabolism in a variety of physiological and pathological processes. Recent studies in humans and mice confirmed that PI3K–Akt signaling plays a crucial role in the regulation of GC growth and apoptosis during follicular development [16]. Research has shown that the PI3K–Akt signaling pathway can regulate the cell cycle and survival of bovine granulosa cells [17]. During mouse follicular development, the PI3K signaling pathway can control the activation of primordial follicles through *FOXO3* in the FOXO family [18]. *FOXO3* can regulate insulin signaling [19,20], and [21] reported that insulin signaling affects the proliferation and differentiation of ovary granulosa cells in mice through FOXO3 phosphorylation. Insulin exerts a gonadotropin effect in the ovaries, and this effect is mediated by interactions between the respective signaling pathways at key nodes, such as the MAPK and AKT pathways [22]. The PI3K–AKT–FOXO signaling pathway is a central pathway that controls the growth and metabolism of all cells [23]. An increasing number of studies demonstrate that the PI3K–AKT–FOXO3 signaling pathway and insulin secretion pathway are closely related to ovarian function and that the PI3K–Akt signaling pathway plays crucial roles in follicular development.

*WNT2* is expressed in rat ovary granulosa cells at all stages of follicular development [24]. In addition, *WNT2* regulates gap junction signaling in mouse folliculogenesis [25]. In this study, *WNT4* was identified as a DMG. Throughout the entire follicular development process, *WNT4* is also expressed in mouse granulosa cells [26]. *WNT4* deletion in granulosa cells in mice results in sub-fertile females with healthy fetal follicles and small ovaries [27]. In addition, *WNT4* plays a regulatory role in embryonic gonadal function and male gonadal development in mice [28]. Studies indicate that *WNT4* plays an important role in follicular maturation and that the WNT2- and WNT4-induced activation of the Wnt signaling pathway promote granulosa cell proliferation [27]. The Wnt signaling pathway is an important pathway that regulates follicular development.

It has been shown that the Notch1 receptor in the Notch signaling pathway promotes follicular maturation in mice [29]. In mice, *Notch2*, *Notch3*, and Jagged 2 are expressed in developing follicular granulosa cells [30]. *Notch2* is a key member of the Notch signaling pathway and potentially plays a role in ovine follicle development by regulating the growth of granulosa cells [31]. Studies have shown that granulosa cell proliferation depends on the Notch signaling pathway [32] and that Notch signaling plays a crucial role in early follicular formation in chickens [33]. The Notch signaling pathway has been shown to be essential for follicular development and fertility [34]. Therefore, the KEGG enrichment results suggest that the identified DMGs are associated with the process of ovarian follicular development.

The association analysis between MeRIP-seq and RNA-seq resulted in 70 differentially expressed and methylated genes. Six DMGs (*WNT4*, *FN1*, *FGF16*, *CRISPLD2*, *AMH*, and *HEY1*) and four DEGs (*GNAQ*, *FGF16*, *AMH*, and *WNT4*) were selected for validation based on network analysis of candidate genes. However, differences were found between the validation results of the DEGs and the transcriptome data (Figure 7B,C). Owing to the ability of m^6^A to regulate mRNA stability, we hypothesize that this result may be due to the influence of m^6^A modification on mRNA expression levels. We investigated the relationships between these seven genes and follicular development. According to reports, *FN1* is a member of the FN family and is vital to follicular development [35]. In addition, *FN1* is enriched in the PI3K–Akt signaling pathway, which is closely related to follicular development. *WNT4* plays an important role in the maturation of follicles. The authors of [36] reported that the *FGF16* gene may play a key regulatory role in ovary development, especially in oocyte growth as well as in Nile tilapia *Oreochromis niloticus*. *AMH* is secreted by the granulosa cells of secondary follicles and plays an important role in the recruitment of ovary primordial follicles and the selection of dominant follicles in mouse and rat ovaries [37]. DMG *HEY1* is enriched in the Notch signaling pathway and is a downstream effector of Notch [38]. These findings indicate that the Notch signaling pathway plays a crucial role in follicular development. In summary, we found that key DEGs play important roles in follicular-related reproductive processes.

Some studies have shown that egg production is determined by ovary function [38]. The egg production performance of laying hens is closely related to ovary and follicular development, and the development status of the follicles has a direct effect on the egg numbers of chickens [1]. Successive follicle selection is important for egg production and reproductive performance in chickens [2]. The authors of [38] identified *WNT4* and *AMH* as candidate genes associated with egg production in chickens. *WNT4* and *AMH* are also key genes we identified. Seven key genes with m^6^A modifications were enriched in pathways related to ovary and follicle development, which affects the egg production process. The results of the present study revealed that chickens with higher levels of m^6^A methylation have significantly greater egg production than those with lower levels of modification. This genetic information will help elucidate the molecular mechanisms of egg production. Overall, we revealed the importance of these key genes in controlling relay, and demonstrated that these genes have a significant effect on egg production. Our study demonstrated that m^6^A modification is involved in the egg production process in Gushi chickens; however, this study is based on omics and has only been validated at the m^6^A and mRNA levels. The functions of the key genes and the specific molecular mechanisms of m^6^A in ovary development and egg production need to be further explored.

## 4. Materials and Methods

### 4.1. Experimental Animals and Sample Collection

The Gushi chicken is a domestically produced dual-purpose chicken cultivated by Henan Sangao Agriculture and Animal Husbandry Co., Ltd. (Xinyang, China) in the core breeding group of the Gushi chicken farm. After 12 weeks of age, the chickens were raised in separate cages and allowed to drink water freely. Chickens were raised, managed, and vaccinated according to the normal management standards for Gushi chickens. A closed chicken coop had a temperature of 25–28 °C, 40–70% humidity, good ventilation, and breathability, providing a suitable breeding environment.

First, individual egg production statistics were assessed using 764 Gushi chickens from the same batch until they reached 43 weeks of age. In the process of counting individual eggs, the numbers of eggs produced at different stages were classified and analyzed, and the eggs produced were grouped and used to determine the high-yield egg group and the low-yield egg group. According to the grouping criteria of [39], we defined individuals with a total egg number of the top 5% of the population at 20 weeks of age as the H group, and individuals whose EN was 0 as the 20-week-old L group. At 28, 36, and 43 weeks of age, the top 10% of chickens with the highest egg production were selected as the H group, and the bottom 20% of chickens with the lowest egg production were selected as the L group.

According to the statistical results, 85–95% of Gushi chickens were in the H group at 43 weeks, and 45–55% were in the L group. The average egg production rate was 60–65%. The average total EN of H group Gushi chickens was 125.83 ± 5.53, which was significantly greater than the average EN of this population (94.36). The average total EN of the L group was 79.00 ± 2.53, which was significantly lower than the average EN of this group (94.36). At weeks 20, 28, 36, and 43, the chickens were anesthetized using intraperitoneal injection of 40 mg/kg body weight pentobarbital. Ovarian tissue samples were taken from the high-yield and low-yield groups and weighed. After performing sample collection, the samples were immediately immersed in liquid nitrogen for freezing and stored in a −80 °C freezer. Ovarian samples were collected from each chicken within ten minutes after euthanasia, and all sample collection was completed within an hour.

### 4.2. RNA Isolation, Library Construction and Sequencing

Total RNA was isolated and purified from each ovary sample using Trizol reagent according to the manufacturer’s instructions. The quality and quantity of total RNA were determined with an Agilent Bioanalyzer 2100 and an RNA 6000 Nano LabChip Kit (Agilent, Santa Clara, CA, USA) with a RIN > 7.0. Poly(A) RNA was isolated from total RNA via an Arraystar Seq-Star™ Poly(A) RNA Isolation Kit (Arraystar, Rockville, MD, USA). The RNA was fragmented using RNA Cleavage Reagent (Sigma, St. Louis, MO, USA), and the average length of the fragments was 100 nt. The RNA was split into two portions. One set of RNA was used as an input control (no immunoprecipitation experiments were performed). Subsequently, the resulting transcriptome was sequenced directly to generate a library, which was used to eliminate the background during the process of collecting fragments with methylation. The other set of RNA was enriched with an m^6^A-specific antibody. After the capture of m^6^A-modified RNA, the antibody was eluted with magnetic beads to reduce the background noise of nonspecific binding. Strand-specific libraries were constructed for each of the two RNAs. After library construction was completed, the quality of the libraries was tested. The quality of all the libraries was measured using an Agilent Bioanalyzer 2100 system (Agilent Technologies, Inc., Santa Clara, CA, USA). The quality-checked libraries (Gene Denovo Biotechnology Co., Ltd., Guangzhou, China) were subsequently sequenced on an Illumina NovaSeq 6000 platform (San Diego, CA, USA).

### 4.3. Bioinformatics Analysis Process

Reads containing splices, duplicate sequences, adapter contaminants, low-quality sequences, and unidentified bases were removed using FASTP software (v0.19.3) [40], and clean reads were obtained. Clean reads were compared to the ribosomal database of the species using bowtie2 [41], and the unmapped reads were retained for subsequent transcriptome analysis. Clean reads were mapped to the *Gallus gallus* (chicken) reference genome using HISAT2 (v2.1.0) [42]. Peak calling and differential peak analysis were performed on a genome-wide scale via the R package exomePeak2 (v1.5.0) [43] on a grouped basis. IGV software (v2.16.0) [44] was used for integrated visualization of various types of genomic data. MEME (v5.3.3) [45] and HOMER (v4.10) [46] software were used to detect the significant sequence motif in the transcript sequence associated with peaks and perform motif analysis. We performed RNA methylation rate difference analysis on all the peaks in the comparison group using exomePeak2 and screened for different peaks under the conditions of *p* value < 0.05 and |FC| > 1.5. On the basis of the HISAT2 results comparison, we reconstructed the transcripts using Stringtie (v2.1.2) [47] and calculated the expression levels of all of the genes in each sample using RSEM (v1.3.1) [48]. Intergroup RNA differential expression analysis was performed using DESeq2 software (v1.22.2), and the adjusted screening conditions for differentially expressed genes or transcripts were FDR < 0.05 and |FC| > 1.5. GO and KEGG pathway enrichment analyses were performed using KOBAS 3.0, with *p* < 0.05 considered statistically significant. Interaction relationships in the STRING [49] protein interaction database (http://string-db.org, accessed on 21 January 2023) were applied for the analysis of differential gene protein interaction networks. The set of DEGs was extracted from the database, and an interaction network diagram was constructed using Cytoscape (v.3.9.1) [50].

### 4.4. Colorimetric Method

Total m^6^A was detected using EpiQuikTM m^6^A RNA Methylation Quantification Kit (Colorimetric; Amyjet Scientific, Wuhan, China) using total RNA extracted from the ovary tissues of Gushi chickens in the 43 w L and H groups according to the test protocol given by the manufacturer. Briefly, Binding Solution (BS) was added to the assay wells, and then 200 ng of extracted total RNA was added to bind RNA to the assay wells, incubated at 37 °C for 90 min, washed and Capture Antibody (CA) was added, incubated at room temperature for 60 min, washed, and Detection Antibody (DA) was added, incubated at room temperature for 30 min. After removing DA, Enhancer Solution was added and incubated for 30 min at room temperature. After washing, the absorbance (OD450) of each well was read at 450 nm using an enzyme marker. The absolute amount of m^6^A in each sample was calculated using the standard curve generated from the absorbance plots of Positive Control and Negative Control.

### 4.5. Quantitative Real-Time PCR

Six DMGs and four DEGs were selected for verification of their enrichment of m^6^A and relative mRNA expression in the ovarian tissues of 43-week-old Gushi chickens. Total RNA was extracted using an RNA simple total RNA extraction kit (TianGen Biotech, Beijing, China) according to the manufacturer’s instructions, and the RNA concentration and purity were subsequently detected using a NanoDrop 2000 spectrophotometer (Thermo, Waltham, MA, USA). The mRNA was reverse transcribed to cDNA using HiScript III-RT Super Mix (with gDNA wiper) (Vazyme, Nanjing, China). Quantitative real-time PCR was performed using ChamQ Universal SYBR qPCR Master Mix (Vazyme, Nanjing, China) and a Light Cycler 96 q-PCR system (Roche, Basel, Switzerland). The total reaction volume was 10 μL, containing 5 μL of 2 × Taq PCR Master Mix, 3.2 μL ddH_2_O, 0.4 L of each primer (1 M), and 1 μL cDNA. All reactions were performed in duplicate and repeated at least three times independently with the following procedure: an initial cycle at 95 °C for 5 min, followed by 35 cycles of 10 s at 95 °C, 10 s at 60 °C and 30 s at 72 °C, and a final cycle at 72 °C for 1 min. The mRNA expression was calculated by the 2^−ΔΔCt^ method [51]. The expression of the proteins was normalized to that of *GAPDH*. Detailed primers are shown in Supplementary Table S3.

### 4.6. Methylated RNA Immunoprecipitation (MeRIP)-qPCR

A Total RNA Extraction Kit (TianGen Biotech, Beijing, China) was used to extract total RNA from the ovary tissue of 43 w Gushi chickens using the Ribo MeRIP™ m^6^A Transcriptome Analysis Kit (10 assay) (RN: R11096.6, RiboBio, Guangzhou, China) to perform MeRIP analysis of RNA according to the manufacturer’s instructions. First, RNA was fragmented to approximately 200 nt, followed by the preparation of anti-m^6^A magnetic beads. Subsequently, 1/10 of the total amount was taken as the input group, and the remaining RNA fragments were immunoprecipitated. A Magen Hipure Serum/plasma miRNA kit (R4317-03, Magen, Guangzhou, China) was used for elution and RNA recovery. The obtained RNA was reverse transcribed, and qRT–PCR was performed to determine the enrichment level of m^6^A in the mRNA.

### 4.7. Statistical Analysis

Statistical analysis was conducted using SPSS 24.0 (IBM, Armonk, NY, USA), and the data are presented as the means ± SEMs (*n* = 3). Student’s t test was used to determine the significance of the difference between two samples. GraphPad Prism 8 software (GraphPad Software, San Diego, CA, USA) was used to calculate the mean values, with error bars indicating the standard error of the mean; * *p* < 0.05, ** *p* < 0.01, *** *p* < 0.001, and ns *p* > 0.05 indicate statistical significance. The differences between the egg production levels, ovary weights, peak numbers of genes, and expression levels of the key genes in the H and L groups were detected using Student’s *t* test (Figure 1, Figure 2D and Figure 7).

## 5. Conclusions

In this study, 2241 DMPs, 2068 DMGs, and 1436 DEGs were screened using RNA-seq and MeRIP-seq analyses. A total of 70 genes exhibited simultaneous changes in m^6^A modification and mRNA expression. Six DMGs (*WNT4*, *FN1*, *FGF16*, *CRISPLD2*, *AMH*, and *HEY1*) and four DEGs (*GNAQ*, *FGF16*, *AMH*, and *WNT4*) were enriched in pathways related to ovary and follicular development and are most likely to play key roles in regulating the egg production process. m^6^A methylation may affect egg production in Gushi chickens by altering the expression of genes related to egg production. This study is the first to construct an m^6^A modification map of ovarian tissue in Gushi chickens, laying the foundation for revealing the molecular mechanism of m^6^A mRNA modification in the egg production process of Gushi chickens and providing a theoretical reference for further research on the regulatory mechanism of ovarian development and egg production in Gushi chickens.

## Data Availability

The data provided in this study can be requested from the corresponding author upon reasonable request.

## Supplementary Materials

The following supporting information can be downloaded at: https://www.mdpi.com/article/10.3390/ijms26041677/s1.

## Abbreviations

m^6^A	RNA N6-methyladenosine
43 w	43 weeks of age
MeRIP-seq	Methylated RNA Immunoprecipitation Sequencing
EN	Egg Number
H	High yield
L	Low yield
qRT-PCR	Quantitative real-time PCR
RNA-seq	RNA Sequencing
IP	Immunoprecipitation
DMG	Differentially Methylated Genes
5′ UTR	5′ Untranslated Regions
3′ UTR	3′ Untranslated Regions
CDS	Coding DNA Sequence
DMPs	Differentially Methylated Peaks
DEG	Differentially Expressed Genes
GSEA	Gene Set Enrichment Analysis
MeRIP	Methylated RNA Immunoprecipitation
BS	Binding Solution
CA	Capture Antibody
DA	Detection Antibody
