# Supplementary material for: Dynamic m6A Modification Landscape During the Egg Laying Process of Chickens
Source: Int J Mol Sci. 2025 Feb 16;26(4):1677. doi: 10.3390/ijms26041677 (PMC11855680; doi:10.3390/ijms26041677)
Supplement: Supplementary file 1 [file ijms-26-01677-s001.zip › Supplementary File S1.pdf]

Table S1. Statistical Table of Base Information.

Sample	CleanData(bp)	AF_Q20(%)	AF_Q30(%)	AF_N (%)	AF_GC (%)
H1_IP	4,616,436,720	4,527,310,980 (98.07%)	4,365,046,040 (94.55%)	107,555 (0.00%)	2,109,528,139 (45.70%)
H1_input	6,077,335,872	5,912,436,561 (97.29%)	5,627,683,580 (92.60%)	138,966 (0.00%)	3,017,682,415 (49.65%)
H2_IP	4,623,322,134	4,546,349,200 (98.34%)	4,398,576,903 (95.14%)	108,218 (0.00%)	2,112,425,699 (45.69%)
H2_input	6,456,429,467	6,319,565,179 (97.88%)	6,066,813,339 (93.97%)	144,938 (0.00%)	3,227,745,220 (49.99%)
H3_IP	4,652,683,065	4,567,647,819 (98.17%)	4,411,568,289 (94.82%)	104,200 (0.00%)	2,140,745,164 (46.01%)
H3_input	6,753,979,972	6,601,670,803 (97.74%)	6,331,411,024 (93.74%)	168,067 (0.00%)	3,382,060,158 (50.08%)
L1_IP	4,710,570,116	4,629,072,491 (98.27%)	4,478,306,450 (95.07%)	108,686 (0.00%)	2,225,876,426 (47.25%)
L1_input	6,518,181,766	6,375,770,909 (97.82%)	6,117,071,500 (93.85%)	152,193 (0.00%)	3,265,511,765 (50.10%)
L2_IP	4,669,712,158	4,581,166,585 (98.10%)	4,424,283,220 (94.74%)	107,032 (0.00%)	2,212,641,098 (47.38%)
L2_input	6,620,435,228	6,481,053,789 (97.89%)	6,227,729,367 (94.07%)	161,150 (0.00%)	3,327,603,910 (50.26%)
L3_IP	4,443,995,371	4,355,945,669 (98.02%)	4,208,245,039 (94.70%)	107,364 (0.00%)	2,115,900,128 (47.61%)
L3_input	6,696,278,049	6,539,074,151 (97.65%)	6259504478 (93.48%)	172,254 (0.00%)	3,350,123,618 (50.03%)

Table S2. Summary of reads mapped to the *Gallus gallus* (chicken).

Sample	Total_Reads	RawDatas	CleanData(%)	Unmapped_Reads	Unique_Mapped_Read	Multiple_Mapped_reads	Total_Mapped
H1_IP	59,379,250	61,316,532	60,173,412 (98.14%)	11,996,840(20.20%)	46,630,682(78.53%)	751,728(1.27%)	47,382,410(79.80%)
H1_input	40,339,040	40,890,708	40,719,144 (99.58%)	2,773,360(6.88%)	36,754,126(91.11%)	811,554(2.01%)	37,565,680(93.12%)
H2_IP	59,126,990	60,772,296	59,769,834 (98.35%)	11,083,267(18.74%)	47,245,510(79.91%)	798,213(1.35%)	48,043,723(81.26%)
H2_input	42,941,610	43,413,722	43,250,800 (99.62%)	2,851,179(6.64%)	39,184,806(91.25%)	905,625(2.11%)	40,090,431(93.36%)
H3_IP	58,977,482	60,756,534	59,769,564 (98.38%)	11,883,778(20.15%)	46,246,199(78.41%)	847,505(1.44%)	47,093,704(79.85%)
H3_input	44,876,326	45,466,938	45,264,844 (99.56%)	3,257,988(7.26%)	40,650,795(90.58%)	967,543(2.16%)	41,618,338(92.74%)
L1_IP	58,106,480	59,843,086	58,973,676 (98.55%)	11,812,113(20.33%)	45,494,017(78.29%)	800,350(1.38%)	46,294,367(79.67%)
L1_input	43,231,434	43,852,430	43,662,628 (99.57%)	2,857,202(6.61%)	39,500,678(91.37%)	873,554(2.02%)	40,374,232(93.39%)
L2_IP	58,451,026	60,087,872	59,042,470 (98.26%)	11,747,848(20.10%)	45854761(78.45%)	848,417(1.45%)	46,703,178(79.90%)
L2_input	44,050,230	44,500,778	44,332,362 (99.62%)	2,861,955(6.50%)	40,244,997(91.36%)	943,278(2.14%)	41,188,275(93.50%)
L3_IP	53,311,464	54,591,624	53757354 (98.47%)	9,453,466(17.73%)	43,005,101(80.67%)	852,897(1.60%)	43,857,998(82.27%)
L3_input	44,567,670	45,053,146	44,864,898 (99.58%)	3,135,458(7.04%)	40,464,414(90.79%)	967,798(2.17%)	41,432,212(92.96%)

Table S3. Details of primer pairs used for the validation

Gene	Primer, 5'-3'	Tm, ℃
METTL3	F: TACGCCGACCACTCCAAACT<br>R: ACGATGGATTGCTCCTTGGC	60
METTL14	F: CGTCGCAGTACAGATGGTGA<br>R: TTGTGAGGGTGGGTCCTACA	60
WTAP	F: CGTATTGCACAGCTTGAGGC<br>R: AGCCTCTCCCTGGTCTGTAG	60
FTO	F: GGTTTCAAGGCGAGCGGTA<br>R: GCATTCTGGCTTCTGCTCTTC	60
ALKBH5	F: TGTCAGGAAGCAACAGGGAC<br>R: AGCTGAAGTTGCTCCGTCTC	60
YTHDF1	F: ACCCTCAGAGACCGAAAGGA<br>R: GTAGACCATGGCGCTTCACT	60
YTHDF2	F: TCCTACTCTCTGGGTGAGGC<br>R: GCGTAATTGCTGCTGTAGCC	60
YTHDF3	F: CAGCGTCGACCAGAGACCTA<br>R: AGTATGGCATTGGAGGGTCAC	60
YTHDC1	F: GATGAGTTGCAGGAGAGCCC<br>R: GCTTTGGCATCATCTGCCTT	60
RBM15	F: TCACCCAACGTCTTCGTCTG<br>R: CCCGCTGTTCTCCTTGTCTT	60
GNAQ	F: CAACGACGAGATCGAGAGGC<br>R: ACTTCCCGCACTAACTGAGC	60
WNT4	F: TGACCACGACCTCAAGAACG<br>R: GTGGATTTCCACCACCCGAT	60
FGF16	F: GAGTTCATCAGCCTGGCAGT<br>R: GTAATGCCTCTCCGAGTCCG	60
AMH	F: AAGATGACCGTGGCTTCTCG<br>R: CATGGAACGGCAGCCTGAA	60
m6A-WNT4	F: CTTCCCCTCTCTAACTGACCCT<br>R: AGCGTCTGGGTCGTTTGCT	60
m6A-FN1	F: TACCACCAGGCCACAAACAC<br>R: GCTCCATCTTGTTTCAAGGCA	60
m6A-FGF16	F: GCGAGGGGTACAGGACTAAG<br>R: TGCAGGTATTTTGGCAGGGT	60
m6A-*CRISPLD2*	F: GCAGCCATAGGGATCTCGAC<br>R: ACGTCCATCAGAGAAGACGC	60
m6A-*AMH*	F: GAACAGGGAGGATGAAGGCG<br>R: GGGTCCCTTTTCTCGGCAG	60
m6A-HEY1	F: CAGCAAACCTTGGCAAACCA<br>R: AGGCTTCCCCACCCTTACTA	60
GAPDH	F: GAACATCATCCCAGCGTCCA<br>R: CGGCAGGTCAGGTCAACAAC	60
