# Supplementary material for: Dynamic m6A Modification Landscape During the Egg Laying Process of Chickens
Source: Int J Mol Sci. 2025 Feb 16;26(4):1677. doi: 10.3390/ijms26041677 (PMC11855680; doi:10.3390/ijms26041677)
Supplement: Supplementary file 1 [file ijms-26-01677-s001.zip › Supplementary File S2.pdf]

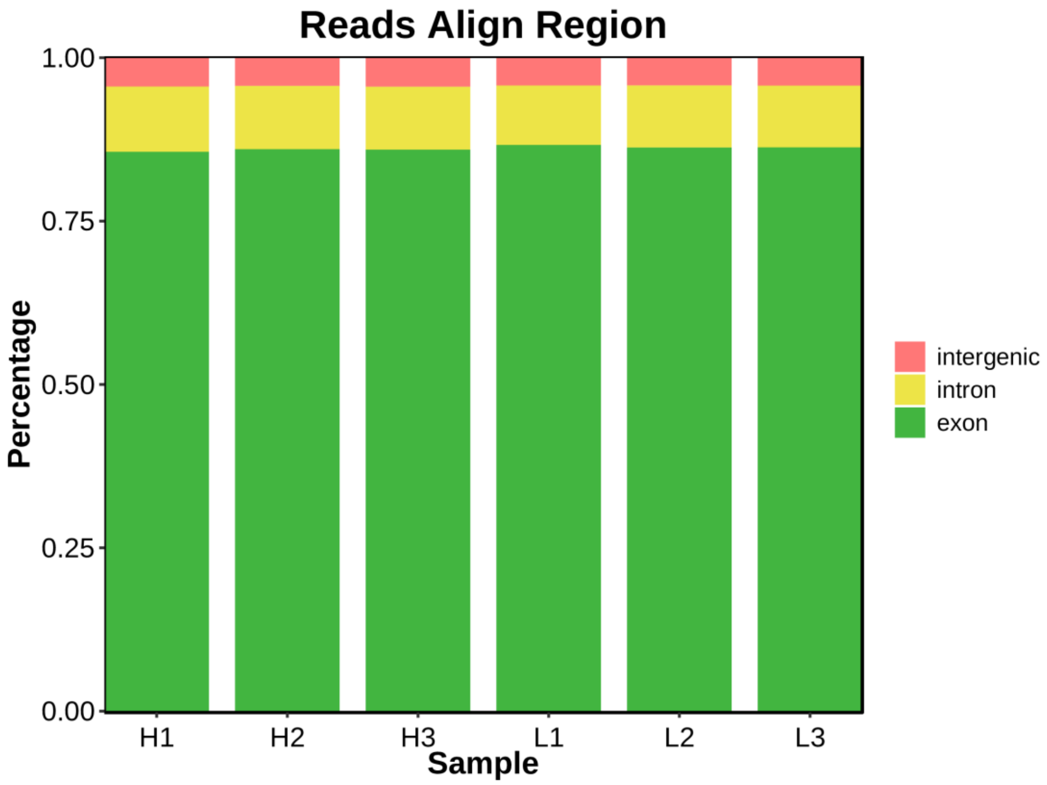

Figure S1. Distribution statistics of reads in the reference genome.

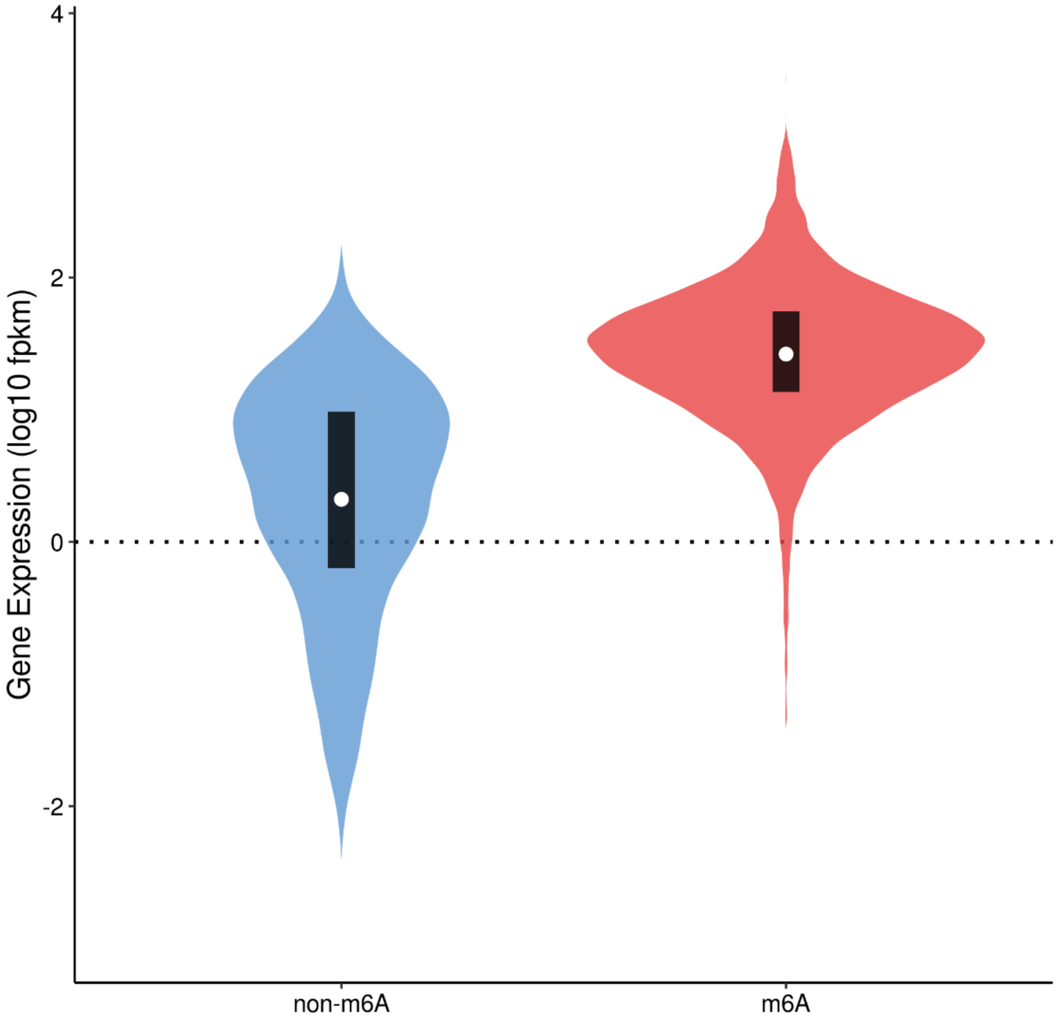

Figure S2. Violin plot of expression with/without m6A modified gene.
